# Chylous Fistula following Axillary Lymphadenectomy: Benefit of Octreotide Treatment

**DOI:** 10.1155/2016/6098019

**Published:** 2016-01-26

**Authors:** Elena González-Sánchez-Migallón, José Aguilar-Jiménez, José Andrés García-Marín, José Luis Aguayo-Albasini

**Affiliations:** ^1^General Surgery Department, Morales Meseguer University Hospital, Mare Nostrum International Excellence Campus, Murcia University, 30008 Murcia, Spain; ^2^Servicio de Cirugía General del Hospital General Universitario “JM Morales Meseguer”, Avenida Marqués de los Vélez s/n, 30008 Murcia, Spain

## Abstract

Chyle leak following axillary lymph node clearance is a rare yet important complication. The treatment of postoperative chyle fistula still remains unclear. Conservative management is the first line of treatment. It includes axillary drains on continuous suction, pressure dressings, bed rest, and nutritional modifications. The use of somatostatin analogue is well documented as a treatment for chylous fistulas after neck surgery. We present a case of chylous fistula after axillary surgery resolved with the use of octreotide.

## 1. Introduction

Nowadays axillary lymphadenectomy is a basic procedure for the locoregional treatment of breast cancer. Chylous leakage after axillary lymph node dissection is infrequent with an incidence of less than 0.5%. Chyle fistula can cause extreme morbidity because of the loss of fluids, electrolytes, and other nutrients. The majority of chyle leaks respond to conservative management.

We present a single case of axillary chylous fistula recorded in our Breast Unit and resolved with the use of octreotide.

## 2. Case Presentation

**Figure 1:**
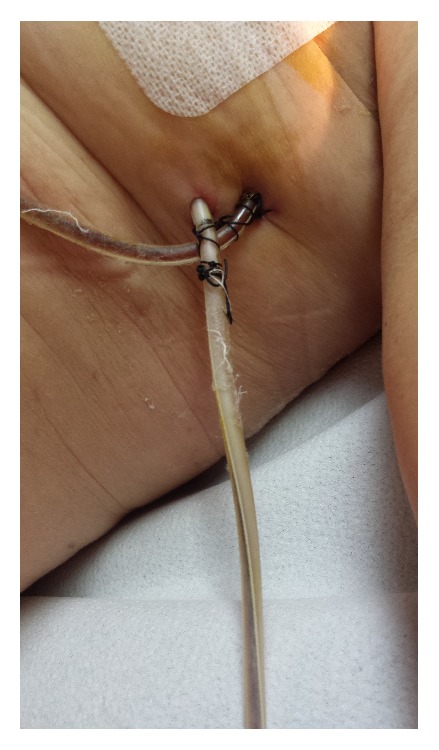


A 72-year-old woman consulted for a nodule in the left breast. The usual diagnostic protocol was carried out, including mammography, ultrasonography with core-needle biopsy of the nodule, and axillary fine-needle aspiration biopsy. Following a negative extension study and a diagnosis of cT2cN2M0 invasive ductal carcinoma, the patient underwent a left-sided modified radical mastectomy. She was discharged on the second day postoperatively with no apparent complications and with drains inserted for ambulatory use. Outpatient follow-up on the tenth day postoperatively revealed the presence of a milky fluid in the axillary drain and a serohaematic fluid in the subcutaneous drain (Figure 1). A cytological, microbiological, and biochemical study of the drainage fluid yielded the following: 2700 leukocytes/dL, 162 mg/dL glucose, 28 g/L proteins, 79.8 UI/L LDH, 139.56 mg/dL cholesterol, and 38 mmol/L (1330 mg/dL) triglycerides. A diagnosis of axillary chylous fistula was established and treatment was implemented including medication with octreotide (0.1 mg/8 h subcutaneously). No nutritional modifications were done but the drainage device and pressure dressing remained. There was a steady decrease in leakage and in triglyceride concentration on serial analysis and the milky appearance disappeared. The drains were removed 12 days after diagnosis of the chylous fistula and 9 days after the start of treatment.

## 3. Discussion

Lymphatic fistula is an uncommon but severe complication that may appear after thoracic, abdominal, or neck operations, but seldom after axillary surgery. Axillary lymph node dissection remains an integral part of surgical treatment in patients with breast cancer. The most usual complications are seroma, chronic lymphoedema, and sensitive alterations on the inner arm. Axillary chylous fistula is a complication with an incidence of less than 0.5%, according to authors such as Nakajima or Singh, unlike chylous fistula after neck dissection, which has an incidence of 1–3% [1–4].

Chylous fistula originates from a lesion in the lymphatic vessels which causes leakage of lymphatic fluid to the thoracic or abdominal cavity or to the exterior as an external fistula. It was first described in the 17th century following accident-related trauma but today usually manifests as a postoperative complication [5]. Low-output fistulas are considered to be those with a leakage of <500 mL/24 h and high-output fistulas those that produce >500 mL/24 h. However, the importance of these fistulas lies in their capacity to compromise the patient's nutritional and immunological balance, delay the healing of surgical wounds, and lengthen hospital stay [5, 6].

The thoracic duct is the common final route for most lymphatic fluid, enabling it to return to the blood flow. The thoracic duct usually drains to the internal jugular vein or subclavian vein but it may join the external jugular vein or innominate vein [4, 5, 7]. Classically, the cause of chylous fistula is assumed to be injury to the main thoracic duct or its terminal rami [2–4, 8, 9]. However, an increasing number of authors suggest that the injury is more probably caused at the subclavian duct or its tributaries, as this duct is very inconsistent and its location is closely related to Berg's level II lymph node dissection [3, 6]. The risk factors for the development of axillary lymphatic fistula obviously include surgical technique, but also obesity and too early exercise of the shoulder [4].

Diagnosis is clear from the presence of a milky output in the drainage contents [5] and is confirmed by a triglyceride level of more than 35 mg/dL.

A fistula detected intraoperatively must be repaired immediately with direct ligation of the injured duct or with fibrin sealants or cyanoacrylate adhesives [5]. However, there are multiple options for treatment of a chylous fistula detected in the postoperative period, although it must always begin with conservative management [5, 10]. The first line of treatment aims to reduce fistula flow using a diet low in fat and rich in medium-chain triglycerides, which are absorbed directly into the portal system and avoid the lymphatic system. These measures also include maintaining axillary drainage, a pressure bandage, and rest. Some authors have suggested the use of parenteral nutrition although this measure is very controversial [2–4, 7].

Most chylous fistulas respond well to conservative treatment and close up in a few days. However, other more aggressive forms of treatment should be considered in the case of high-output fistulas (>500–600 mL/24 h) which persist for several days despite conservative measures or in the case of an extremely high output (>2 L) [3, 5, 7, 11]. On such rare occasions it is necessary to reoperate in order to ligate the affected lymphatic vessels or perform a lymphangiography and embolisation of the injury.

The use of octreotide is well documented as a treatment for the more common chylous fistulas of the neck following left-sided lymphadenectomies. The mechanism by which this somatostatin analogue can reduce lymphatic flow is not fully known. It is assumed that the drug causes less intestinal fat absorption and consequently less flow to the damaged duct, which would make it easier to repair with the usual healing process [5]. The normal doses are 6 mg of somatostatin daily in continuous infusion or 0.1 mg/8 h of octreotide subcutaneously [5, 6]. Octreotide has also been recommended for chylous fistula treatment after axillary surgery but as far as we know this is the first case documenting a decrease in triglyceride concentration parallel to a reduction in fistula flow that correlates with the commencement of the drug and corroborates its supposed mechanism of action.

## References

[1] Lee E. W., Shin J. H., Ko H. K., Park J., Kim S. H., Sung K.-B. (2014). Lymphangiography to treat postoperative lymphatic leakage: a technical review. *Korean Journal of Radiology*.

[2] Flores E. V., de Castro G., Casal E., Sobrino C. (2014). Chylous fistula following axillary lymphadenectomy. *Cirugia Espanola*.

[3] Singh M., Deo S. V. S., Shukla N. K., Pandit A. (2011). Chylous fistula after axillary lymph node dissection: incidence, management, and possible cause. *Clinical Breast Cancer*.

[4] Sakman G., Parsak C. K., Demircan O. (2007). A rare complication in breast cancer surgery: chylous fistula and its treatment. *Acta Chirurgica Belgica*.

[5] Jain A., Singh S. N., Singhal P., Sharma M. P., Grover M. (2015). A prospective study on the role of octreotide in management of chyle fistula neck. *The Laryngoscope*.

[6] Rico Arrastia A., Vicente García F., Pérez Omeñca F., Artieda Soto C., Sanz de Pablo M. A., Domínguez Cunchillos F. (2014). Chylous fistula after lymphadenectomy in breast cancer. *Anales del Sistema Sanitario de Navarra*.

[7] Purkayastha J., Hazarika S., Deo S. V., Kar M., Shukla N. K. (2004). Post-mastectomy chylous fistula: anatomical and clinical implications. *Clinical Anatomy*.

[8] Langford R. J., Daudia A. T., Malins T. J. (1999). A morphological study of the thoracic duct at the jugulo-subclavian junction. *Journal of Cranio-Maxillo-Facial Surgery*.

[9] Van Pernis P. A. (1949). Variations of the thoracic duct. *Surgery*.

[10] Taylor J., Jayasinghe S., Barthelmes L., Chare M. (2011). Chyle leak following axillary lymph node clearance—a benign complication: review of the literature. *Breast Care*.

[11] Chow W. T. H., Rozen W. M., Patel N. G., Ramakrishnan V. V. (2015). Chyle leak after axillary lymph node dissection. *Journal of Plastic, Reconstructive and Aesthetic Surgery*.

